# A Unique Kelch Domain Phosphatase in *Plasmodium* Regulates Ookinete Morphology, Motility and Invasion

**DOI:** 10.1371/journal.pone.0044617

**Published:** 2012-09-05

**Authors:** Nisha Philip, Heli J. Vaikkinen, Laurence Tetley, Andrew P. Waters

**Affiliations:** Wellcome Trust Centre for Molecular Parasitology, Institute of Infection, Immunity and Inflammation, Sir Graeme Davies Building, University of Glasgow, Glasgow, United Kingdom; Centro de Pesquisas René Rachou, Brazil

## Abstract

Signalling through post-translational modification (PTM) of proteins is a process central to cell homeostasis, development and responses to external stimuli. The best characterised PTM is protein phosphorylation which is reversibly catalysed at specific residues through the action of protein kinases (addition) and phosphatases (removal). Here, we report characterisation of an orphan protein phosphatase that possesses a domain architecture previously only described in *Plantae*. Through gene disruption and the production of active site mutants, the enzymatically active Protein Phosphatase containing Kelch-Like domains (PPKL, PBANKA_132950) is shown to play an essential role in the development of an infectious ookinete. PPKL is produced in schizonts and female gametocytes, is maternally inherited where its absence leads to the development of a malformed, immotile, non-infectious ookinete with an extended apical protrusion. The distribution of PPKL includes focussed localization at the ookinete apical tip implying a link between its activity and the correct deployment of the apical complex and microtubule cytoskeleton. Unlike wild type parasites, *ppkl^–^* ookinetes do not have a pronounced apical distribution of their micronemes yet secretion of microneme cargo is unaffected in the mutant implying that release of microneme cargo is either highly efficient at the malformed apical prominence or secretion may also occur from other points of the parasite, possibly the pellicular pores.

## Introduction

Malaria is caused by the protozoan *Plasmodium* and is a major health problem in the developing world resulting in over a million deaths annually [1]. The life cycle of the Plasmodium parasite consists of three distinctive developmental stages in the mammalian liver and erythrocytes, and the mosquito vector. During development, the parasite is subjected to diverse host environments, undergoes complex morphological changes and exhibits significant variation in shape, size and motility. Parasite development and the rapid responses to host environment are co-ordinated by cell signalling networks, many of which involve protein phosphorylation.

Reversible protein phosphorylation by protein kinases and phosphatases is implicated in a number of essential eukaryotic processes, including responses to external stimuli and internal processes involving cell proliferation and differentiation. Two-thirds of the 23,000 proteins encoded by the human genome are regulated by phosphorylation highlighting the universality of this post-translational modification [2]. In the malarial parasite, two recent global phosphoproteomic surveys of blood stage asexual parasites identified 1673 and 650 phosphoproteins, corresponding to 30% and 12% of the predicted parasite proteome respectively [3], [4]. Studies have also implicated plasmodial kinases in regulating fundamental cellular processes such as splicing [5], [6], ubiquitination [7], vesicle transport [8] and translational control [9]. Additionally, systematic reverse genetics analyses of protein kinases in both *P. falciparum* and *P. berghei* revealed approximately 50% of the kinome is essential for asexual blood stages and, another 14 kinases are exclusively required during sexual development [10].

Although the pathology of malaria is caused by the asexual stages in the blood stream, transmission into the mosquito vector requires sexual stage development. Functional analyses have identified protein kinases as key regulators at several stages during sexual development. Gametocytes are taken up in a blood meal, where conditions in the mosquito midgut trigger formation of male and female gametes requiring a cGMP dependent protein kinase [11]. The male gamete undergoes three rounds of DNA replication dependent upon the activity of a Ca^2+^ dependent protein kinase (CDPK4) [12], followed by mitogen activated protein kinase (MAP2) regulated cytokinesis and release of microgametes [13], [14]. Fertilization forms a diploid zygote that undergoes meiosis requiring two maternal lineage NIMA-like kinases, NEK2 and NEK4 [14], [15], [16]. Within 12–24 hours the zygote transforms into a polarized motile ookinete, whose motility is regulated by cGMP and Ca^2+^ signalling [17], [18]. The ookinetes penetrate the mosquito midgut wall and transform into oocysts, which over a period of two weeks release sporozoites to invade salivary glands. The developmental stages from oocyst to salivary gland sporozoites require the activity of six kinases [10].

The majority of functional studies on protein phosphorylation in *Plasmodium* have focussed on protein kinases and the study of phosphatases has generally been restricted to molecular and biochemical analysis. Although *Plasmodium* protein kinases are well regarded as effective drug targets [19], studies suggest inhibiting phosphatase activity also has antimalarial effects [20]. Two studies identified 27 protein phosphatases in the Plasmodium genome and, the proteins could be classified into the three major classes namely PPP (phosphoprotein phosphatase), PPM (metallo-dependent protein phosphatase) and PTP (protein tyrosine phosphatase) [21], [22]. Interestingly four phosphatases had no orthologues in the vertebrate host making them excellent targets of therapeutic intervention. One of the unique enzymes is an unusual PPP phosphatase, with a kelch repeat containing N-terminus and a C-terminal PP1-like phosphatase domain (PPKL: protein phosphatase with kelch-like).

The kelch motif normally occurs as a series of four to seven repeats forming a β- propeller tertiary structure and, can be present either at the C or N-terminus [23]. The motif is evolutionarily widespread and implicated in diverse cellular activities including protein degradation (F-box), transcriptional regulation (Keap1), cytoskeletal organization (α-Scruin) and establishment of cell polarity (Tea1p) [24], [25], [26], [27]. Additionally kelch-repeat proteins have been identified as modulators of the cAMP signalling pathway by regulating Ras and Protein kinase A activity [28], [29]. Although kelch-repeat proteins have been identified and characterised in diverse organisms including viruses, fungi, plants and animals, the PPKL enzyme family is detected only in alveolates and plants [21]. The only well characterised PPKL is the Arabidopsis enzyme, BSU1 (*bri1* suppressor), which plays a key role in steroid signalling and is highly expressed in elongating tissue [30]. In *P. falciparum,* PPKL (initially named PfPPα) mRNA expression was detected only in gametocytes indicating a role in sexual stage development [31].

This study demonstrates that *Plasmodium* PPKL performs a critical role during sexual stage development where it maintains the microtubule network and, is crucial for conserving the distinctive morphology of the ookinete. Development of the highly polarized ookinete requires the formation of an apical microtubule organizing centre (MTOC) from which microtubules originate and run along the length of the cell [32]. The sub-pellicular microtubular network is a key component of the ookinete cytoskeleton and is responsible for maintaining the parasite’s shape, flexibility and structural integrity. Moreover, the microtubules regulate organelle transport including micronemes, which carry proteins essential for motility and invasion [33]. Here we also establish that the phosphatase regulates the apical distribution of the invasion apparatus, and parasites lacking PPKL fail to invade the mosquito vector revealing a crucial role for *Plasmodium* PPKL in malaria transmission.

## Results

### P_BANKA 132950 is a Kelch Domain Phosphatase

**Figure 1:**
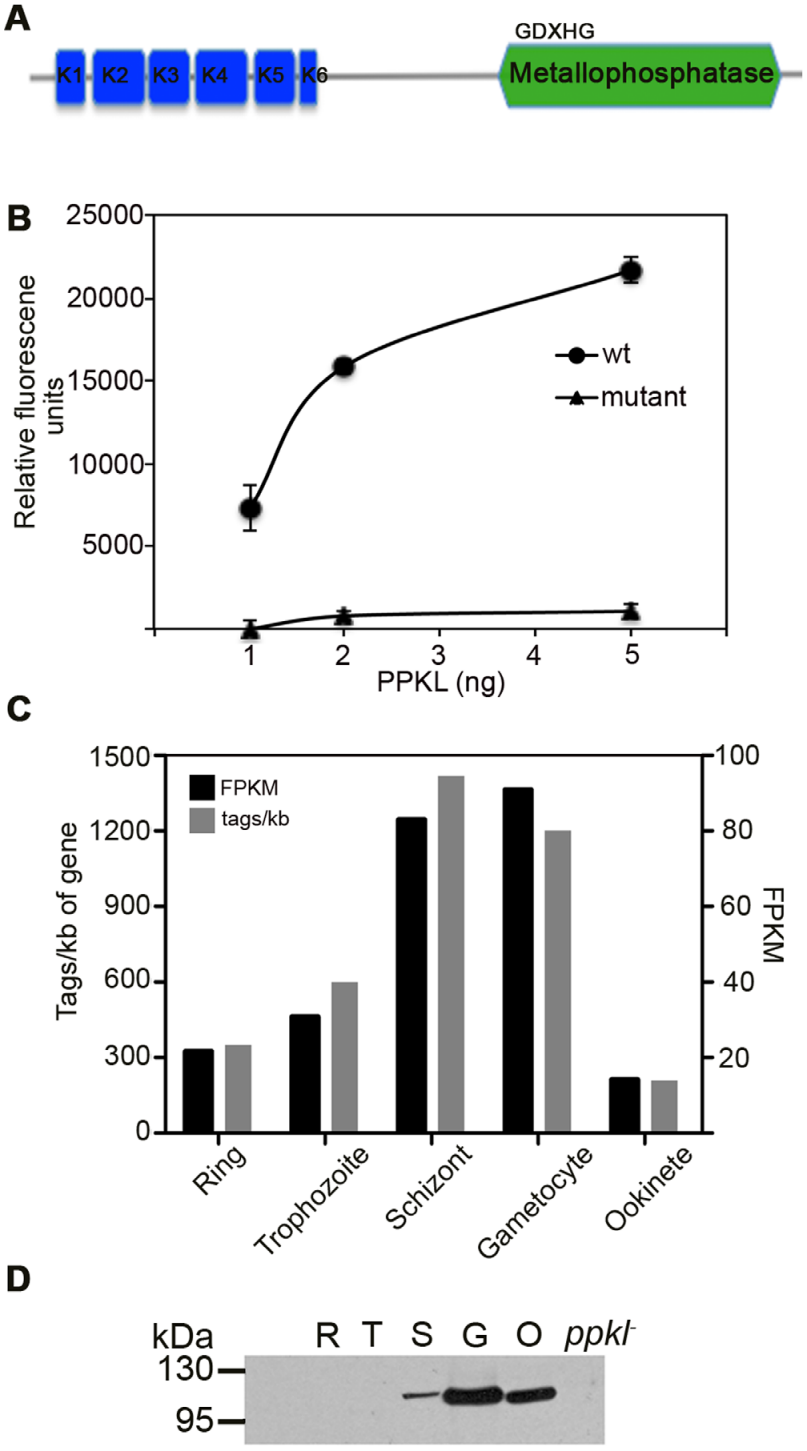
*ppkl* expression and activity. A. Schematic representation of PPKL structure showing N-terminal Kelch repeats and C-terminal catalytic domain. Residues of the first motif of the catalytic domain are indicated B. Phosphatase assay demonstrates PPKL is an active phosphatase whereas the catalytic site mutant (-GAXNE-) shows no activity. C. RNA-Seq data represented as both tags/kb of gene and fragments per kilobase of exon model per million mapped reads (FPKM) indicates *ppkl* mRNA levels are highest in schizonts and gametocytes D. Immunoblots of parasite extracts from rings, trophozoite, schizont stages from a gametocyte non-producer and, gametocyte and ookinete stages using a α-PPKL polyclonal antibody. Protein expression is detected only in schizonts and sexual stages.

The Kelch domain phosphatase family has been identified only in the genomes of Plantae and Alveolates (Figure S1, [21], [34]). All identified members of this family including the Plasmodium PPKL contain a unique conserved structure consisting of N-terminal Kelch repeats followed by a C-terminal phosphoesterase domain (Figure 1A, [34]). Plasmodium PPKL contains five full and a one half kelch domains (Figure 1A, [21]) which is consistent with other known Kelch domain containing phosphatases. The catalytic domain contains 5 inserts, which are unique only to Alveolates, suggesting a mode of enzyme activity or regulation specific to this phylum (Figure S2, [21]). The C-terminal catalytic domain contains the three signature motifs (-GDXHG-, -GDXVDRG- and –GNHE-) of Phosphoprotein phosphatase (PPP) family (Figure S2A). To determine whether PbPPKL was a functional phosphatase, the catalytic domain of the phosphatase was expressed as N-terminal 6X–His tagged protein. As a negative control a catalytic site mutant changing the –GDXHG- motif to –GAXNG- was also generated thereby removing a metal binding amino acid and, also a catalytic residue. The protein’s ability to catalyze 6,8-difluoro-4-methylumbelliferyl phosphate (DiFMUP) substrate to DiFMU (Molecular Probes) was then examined. Whilst the mutant showed no activity, wild-type PPKL catalytic domain was active demonstrating *Pbppkl* codes for a functional phosphatase (Figure 1B).

### PPKL is Predominantly Expressed during Sexual Stage Development

To establish PPKL expression pattern, we examined mRNA levels in our RNA-seq dataset (W.A.M. Hoeijmakers, A. Religa, C.J. Janse, A.P. Waters, & H.G. Stunnenberg, unpublished data) and protein expression using a polyclonal antibody raised against the PPKL catalytic domain and also C-terminally tagged GFP-PPKL (details of transgenic line in methods and Figure S4F–I). While, PPKL mRNA was found in all asexual blood stages, gametocytes and ookinetes (Figure 1C), PPKL protein expression was detected only in schizonts and sexually differentiated parasites (Figure 1D).

**Figure 2:**
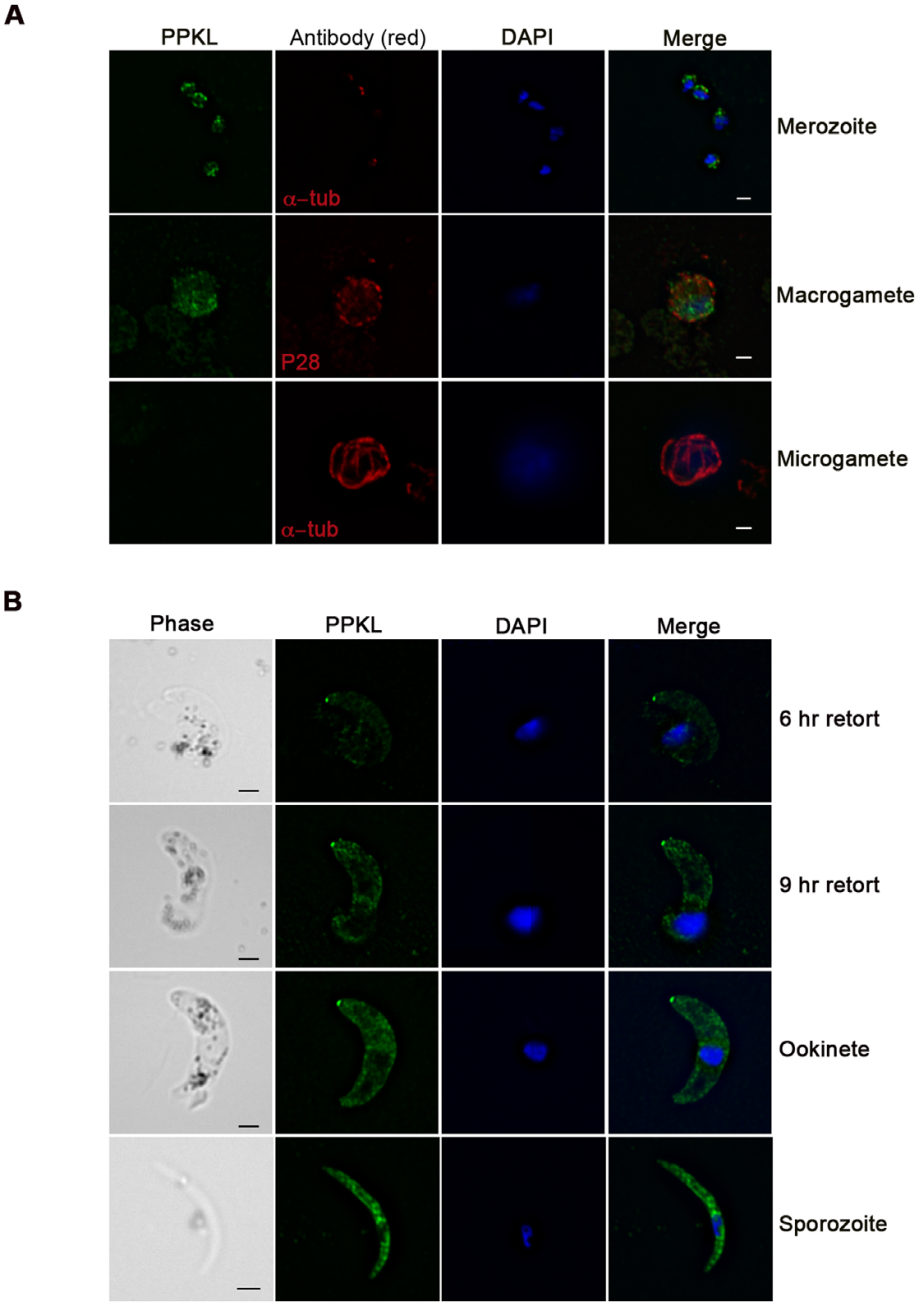
PPKL localization during *Plasmodium* life-cycle. A. PPKL expression in merozoites and activated gametocytes. No expression is observed in the male gametocyte. B. PPKL localization during zygote-ookinete development and in sporozoites. Expression is predominantly cytoplasmic with a focussed expression observed only in emerging tip of the retort and ookinete stages. Bar = 1 µm.

Immunofluorescence studies using anti-PPKL polyclonal antibody showed expression in merozoites, female gametocytes, zygotes, ookinete, and sporozoites (Figure 2, S3). The GFP tagged line showed similar localization (Figure S3A). Interestingly, only female gametocytes expressed PPKL and no protein expression was detected in males (Figure 2,S3B). The phosphatase is localized predominantly in the cytoplasm with a particularly intense expression at the apical tip of the developing and mature ookinete. This distinctive apical expression of PPKL indicates a specific role for the protein at the ookinete’s apical end.

### PPKL is Essential for Maintaining Ookinete Morphology and is Contributed by the Female Gamete

To examine the function of PPKL during the *Plasmodium* life-cycle, the gene was deleted by allelic replacement using a double crossover strategy. (Figure S4A). Targeted integration of the plasmid containing a human dhfr/ts was confirmed by diagnostic PCRs and FIGE, and absence of both transcript and protein was verified by Northern analysis and western blotting (Figure S4B–E).

**Figure 3:**
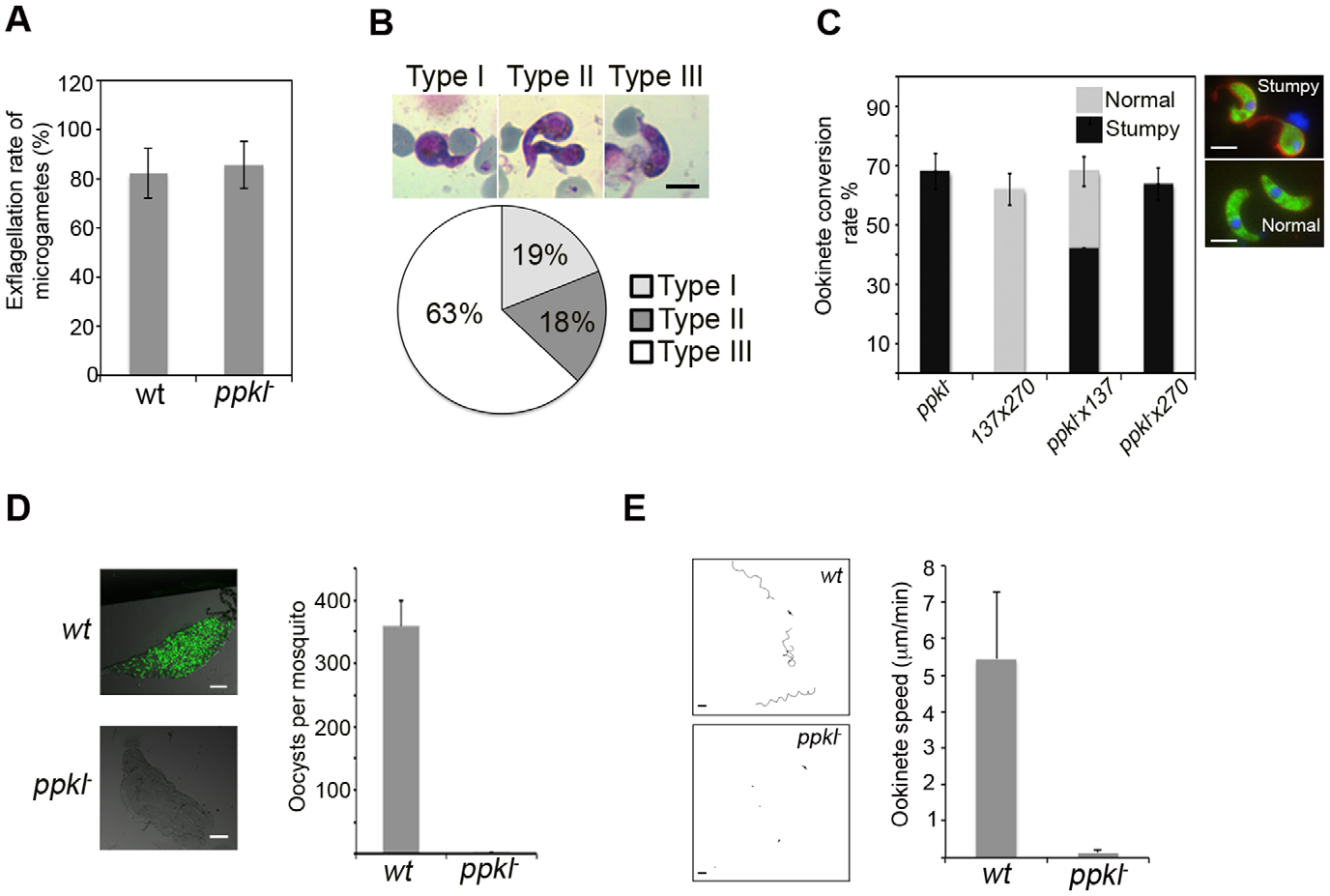
Phenotypic analysis of *ppkl^–^* mutants. A. Exflagellation rates of male gametes from *wt* and *ppkl^–^* parasites (error bar = mean +/− SD, *n = *5, p = 0.73). B. Morphological subtypes of stumpy *ppkl^–^* mutant retorts/ookinetes assessed by Giemsa staining. Pie chart shows the percentage of each subtype within the ookinete population (*n = *3. Bar = 5 µm). C. Ookinete conversion post crosses with *ppkl* ^–^ mutants with only female-fertile (*137*) and male-fertile (*270*) lines. Control is a *137*×*270* cross. Graph shows percentage of fertilized zygotes that converted to retorts/ookinetes (Error bars = mean ±SD; *n* = 3). D. Average number of oocysts per mosquito gut (error bar = mean+/− SD; *n = *16 of wild-type or *ppkl^–^* infected mosquitoes from 1 representative dataset of 5 independent experiments; p<0.0001). Bar = 100 µm. E. Paths of *wt* and *ppkl^–^* ookinetes moving through matrigel over a 20 min period (left panels: bar = 10 µm). Speed of WT ookinetes or *ppkl^–^* mutant retorts 24 hrs post activation of gametocytes (right graph, *n = *15, p<0.0001). See also video S1 and S2.

Further analysis of two independently transfected and cloned *ppkl* deletion mutants demonstrated no growth phenotype during asexual blood stage development or gametocytogenesis. Moreover, gametogenesis of *ppk^–^* male gametocytes assessed by exflagellation rates was identical to wildtype (Figure 3A). However, when cultured *in vitro,* differentiation into ookinete stages was severely impaired producing abnormal forms, with 63% appearing as the Type III form (Figure 3B). Since the effect of PPKL absence was visible post-fertilization it was examined whether the defect was male or female specific by performing genetic crosses between *ppkl^–^* parasites and parasites lines which are specifically male fertile (line 270, *p47^−^*) or female fertile (line 137, *p48/45^−^*). Consistent with the observed wild type pattern of PPKL expression only crosses with wild type females from line 137 generated normal ookinetes (26%) revealing the requirement of PPKL to be female specific (Figure 3C).

### PPKL is Essential for Mosquito Invasion

To test whether aberrant ookinetes could infect the mosquito vector transmission experiments were performed where parasite infected mice were fed to mosquitoes to assess oocyst development. While oocysts developed normally in wild- type parasites, midguts of *ppkl^–^* infected mosquitoes contained no oocysts (Figure 3D).

The defective ppkl^–^ ookinete morphology and its inability to cross the mosquito mid gut epithelium prompted an examination of ookinete motility. Gliding motility was tested for both wild-type and *ppkl^–^* parasites by embedding cultured ookinetes in Matrigel and measuring movement every 20 s over a 20 minute period. While *wt* ookinetes exhibited the typical corkscrew like motion and an average speed of ∼5 µm/min, *ppkl^–^* scarcely moved displaying a slight flexing movement (Figure 3E, Movies SV1 and 2). Taken together these results establish that PPKL is essential for the ookinete’s forward gliding motility and therefore mosquito transmission.

### *Ppkl^–^* Parasites Display Cytoskeletal Defects

**Figure 4:**
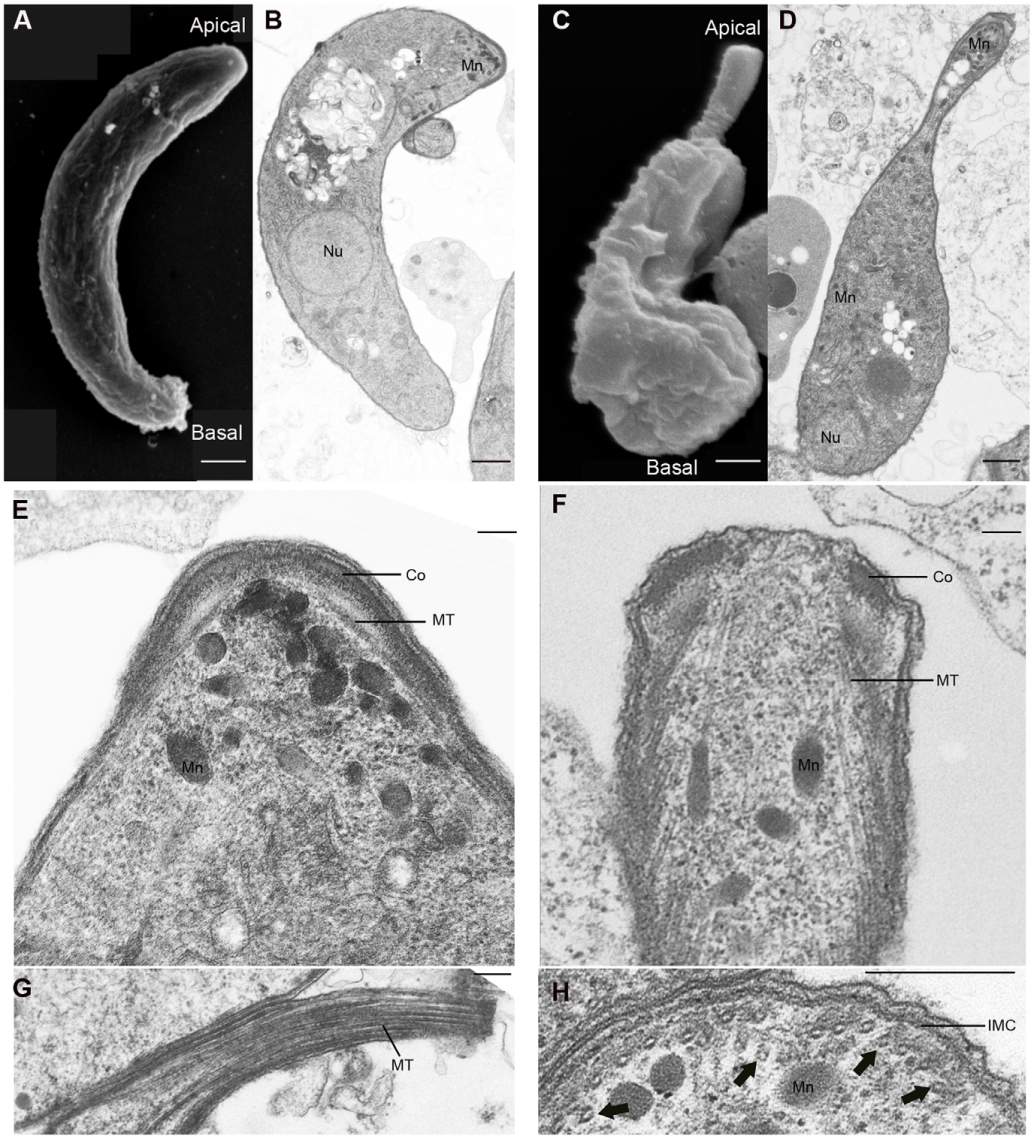
Ultrastructure analysis of wild type and *ppkl^–^* ookinetes. A. Scanning electron micrograph (SEM) of wild type ookinete. B. Transmission electron micrograph (TEM) detailing longitudinal section through wild type ookinete. C. SEM of *ppkl^–^* exhibits an elongated apical and no basal constriction. D. TEM longitudinal section through *ppkl^–^* further shows micronemes dispersed through the cell body. (A–D, bar = 1 µm) E. Detail of wild type apical complex depicts a well-defined collar, attached microtubules and concentrated micronemes. F. Detail of *ppkl^–^* apical end reveals fewer micronemes and an underdeveloped apical complex. G. Longitudinal section of the elongated apical end shows collapsed microtubule bundles H. Loss of contact of microtubules with the IMC (arrowed). (E–F, bar = 0.25 µm). Abbreviations: Micronemes, Mn; Nucleus, Nu; Collar, Co; Microtubules, MT; Inner membrane complex, IMC.

The cytoskeleton and apical complex play critical roles in maintaining the ookinete’s polarity, shape and motility. Due to the mutant ookinete’s gross morphological defects and subsequent inability to move and transmit to the mosquito, ultrastructural analysis was performed to further examine its cytoskeletal organization. Both scanning and transmission EM confirmed the morphological defects observed by light microscopy where the ookinete exhibits no basal constriction and has long apical protrusions (Figure 4A–D). Apicomplexan zoites are enclosed by a pellicle which is a tri-layer membrane consisting of the plasma membrane followed by a double membrane consisting of flattened vesicles named the inner membrane complex (IMC). The organisation of the IMC in mutant ookinetes appeared normal. Motile and invasive stages also possess a highly specialized apical complex, which in ookinetes was composed of the collar, secretory organelles (micronemes) and polar rings. The polar ring forms the microtubule organizing centre (MTOC) of the sub-pellicular microtubules which are essential for maintaining the elongated shape, polarity, and organelle trafficking. Closer examination of the *ppkl^–^* cytoskeleton revealed a significantly less developed apical complex (Figure 4E, F) and fewer micronemes were localized to the apical end (Figure 4D, F). We also observed loss of attachment of the microtubules to the IMC resulting in collapsed bundles of tubules (Figure 4G, H). These data are consistent with a lack of apical organization and disassembled microtubules resulting in aberrant morphology and impaired gliding motility.

**Figure 5:**
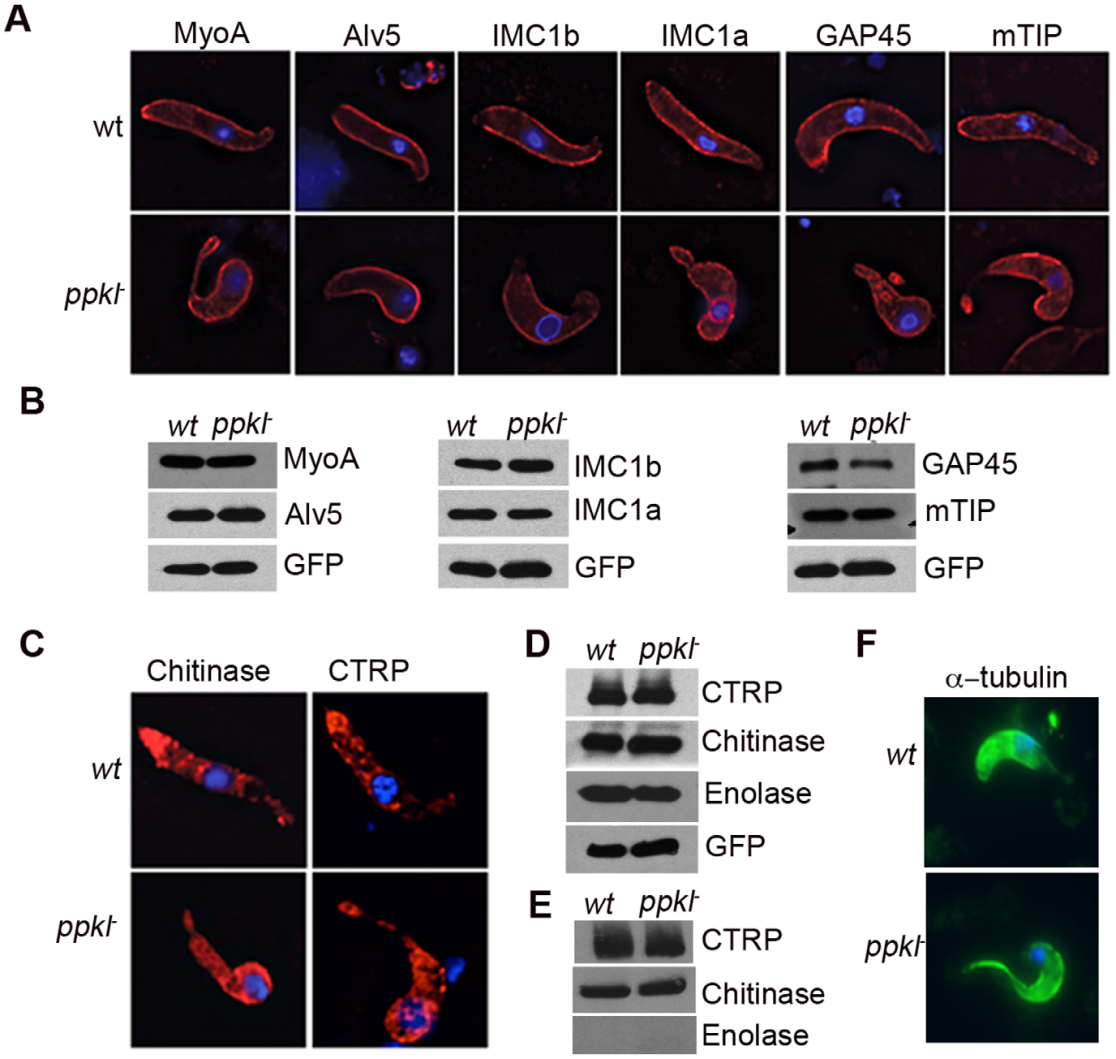
Expression and localization of structural and invasive proteins of the ookinete. A. Direct immunofluorescence detection of alveolar and motor proteins (red) B. Total protein expression of the same proteins; GFP expression was used to normalize loading. C. Micronemal proteins CTRP and chitinase were spread throughout the cell body (red) in the mutant and not localized to the apex. D. Total protein expression of CTRP and chitinase in mutant ookinetes is identical to *wt*. E. Secretion of CTRP and chitinase remains similar to *wt*. Enolase, an abundant non-secreted protein is not detected in the ookinete medium. F. Abnormally intense α-tubulin staining was observed in *ppkl^–^* apical end (green). Ookinetes were processed 24 hour post-activation. The nucleus was counter-stained using DAPI (blue). Bar = 1 µm.

Immunofluorescence and western blotting of IMC proteins suggested no mis-localisation or expression of motor proteins and alveolins in *ppkl^–^* parasites (Figure 5A, B). Although micronemal proteins such as CTRP and chitinase, were spread throughout the cell body rather than concentrated at the apical end which was in agreement with the ultrastructural data, no defects in micronemal secretion were observed (Figure 5C–E). However, further examination of microtubules by immunofluorescence revealed abnormally intense α-tubulin staining at the ookinete’s apical end (Figure 5F). Taken together this data suggests that the disorganization of microtubules, may disrupt ookinete locomotion and prevent it from accessing or penetrating the mid-gut epithelium.

## Discussion

Spatial and temporal regulation of the enzymes catalysing (de)phosphorylation reactions i.e. kinases and phosphatases is crucial for maintaining cell homeostasis. Deregulation of phosphorylation-based signal transduction pathways have been implicated in a variety of disease states including cancer, diabetes, and neurodegenerative diseases, which have led to exploration of these pathways for therapeutic intervention [35]. Although kinases are well established as important signalling molecules and valuable drug targets, the role of phosphatases in cell signalling is less well defined and only recently have they been recognized as viable therapeutic targets [36]. Similarly in *Plasmodium,* employing global analyses, biological roles of a number of kinases have been elucidated [3], [10], but functional studies on *Plasmodium* phosphatases are limited. The *Plasmodium* genome contains only 27 protein phosphatases [22], [37] and is, therefore easily amenable to systematic genetic and functional analysis. Consequently, determining stage specific requirements of these genes would enable rational design of combination therapeutic strategies to target both the symptomatic (blood stages) and transmission (early sexual stages) phases of the parasite life-cycle.

### PPKL Phosphatase is Essential to Ookinete Development

The data presented here demonstrate that the putative *ppkl* gene encodes a catalytically active phosphatase, which functions specifically during sexual stage development. Direct analysis of protein expression confirmed the findings of more global surveys [4], [38] indicating expression in late stage schizonts and all sexual stage parasites. Although PPKL expression was observed in schizonts and female gametocytes, its first point of essentiality in the *Plasmodium* life cycle is post-fertilization in the production of a motile, morphologically normal, infectious ookinete.

### ppkl^–^ Ookinete Apical Organisation is Compromised

Ookinetes are the only zooite produced by *Plasmodium* that develop outside a host cell and furthermore their apical organisation of the ookinete is also unique amongst the *Plasmodium* zoites: the ookinete forms an apical collar and lacks the rhoptries present in other zoites (merozoite and sporozoite) although micronemes are numerous and readily observed. Ookinetes lacking PPKL have fewer apically distributed micronemes which might be anticipated to reduce invasion competence yet secrete wild type levels of the known virulence factors CTRP and chitinase. Furthermore, mutant ookinetes lacking a male-inherited formin-like protein termed MISFIT have demonstrated that an abundant distribution of micronemes to the ookinete apical tip is not a prerequisite for ookinete invasion of the midgut wall [39]. Interestingly, cryofracture EM studies have identified large pores in the pellicle of the ookinete, which might provide alternate routes for trafficking of secretory molecules independent of the microtubule/apical pore system [40]. The nature and mechanism(s) of secretion of microneme cargo in such mutants will be intriguing to elucidate. Apical protrusions and misshapen ookinetes with impaired motility reminiscent of those produced by *ppkl^–^* ookinetes have been observed in a variety of gene deletions targeting IMC components (IMC1 b and IMC1 h (Alv3)) but detailed ultrastructural analysis resulting from these other mutations which still produced infectious ookinetes has not been reported [41], [42]. The authors concluded that it was not possible to directly ascertain if the deformities directly caused the impaired motility which is also true in the case of the *ppkl^–^* mutants. The ability of kelch domain proteins to regulate actin polymerisation and the known regulation of protein components of the gliding motility apparatus by phosphorylation (GAP45 & MTIP by CDPK1) [43] suggest that motility itself might also be directly influenced by PPKL. PPKL may also control the regulators of gliding motility: mutants of calcium-dependent protein kinase 3 [18] and nucleotide cyclase guanylyl cyclase β [43], [44] showed severe defects in ookinete gliding motility and mosquito mid-gut invasion. Given that gross rearrangements of the IMC and gliding apparatus were not observed in ookinetes lacking PbPPKL, whether the phosphatase interacts with any of these effectors remains uncertain. However, electron microscopy revealed various structural abnormalities at the apical end of the *ppkl* ^–^ retort in terms of its microtubule organisation and their association with the IMC, as well as an underdeveloped apical complex. Therefore it is possibly the overall integrity of the apical complex and its contribution to the establishment of a fully functional IMC that contributes most to the non-motile phenotype in the *ppkl^–^* mutant. To our knowledge this is the first study demonstrating a gene essential for the preserving the apical complex. The focused localization of PPKL at the apical end of the retort as early as 6 hours post fertilization implies the phosphatase could play an essential role in establishing and/or maintaining the structural and functional integrity of the apical complex.

### Signalling and PPKL

Signalling events mediated by (cascades of) phosphatases can be crucial to differentiation. In bloodstream African trypanosomes differentiation to the transmissible stumpy form which occurs in the midgut of the Tsetse fly is prevented by the activity of a tyrosine phosphatase, TbPTP1 negatively regulating a second Ser/Thr phosphatase, TbPIP39 [45], [46]. PPKL is known to be regulated by phosphorylation in its guise as BSU1 in *A. thaliana* where one of the kinases that acts upon it, is the constitutive differential growth 1 kinase (CDG1) which itself is activated by the receptor kinase BRI1 upon binding of a brassinosteroid hormone. BSU1 is phosphorylated on three residues where one of the residues, S444, is not only conserved in all *Arabidopsis* BSU1 paralogues [47], [48], but also in PPKL (S480 in PbPPKL). Interestingly, global phosphoproteomic studies in *Plasmodium* demonstrated that PPKL contains at least 9 sites which might be differentially phosphorylated during development (S448, S451, S488 and T873 in schizonts; S409, S416, S436, S480, and T498 in ookinetes, Figure SF2B,C) [4], GeneDb). The most similar *Plasmodium* kinase to CDG1 by BLAST homology is TLK2 (PBANKA_92700) which has been shown to be non-essential by gene disruption [10] indicating that this is unlikely to be the enzyme critical for PPKL activation and emphasising the difference in upstream signalling between *Arabidopsis* and *Plasmodium*. Identification of the signalling cascade for PPKL in *Plasmodium* will ascertain whether the pathway is similar to BR signalling in plants and would be an essential step in elucidating potential transmission blocking drug targets.

In summary we have shown that PbPPKL expression and localization are developmentally regulated and, the phosphatase like many regulatory proteins has stage specific functions. The conservation of this protein throughout Apicomplexans suggests it might be key in maintaining the cytoskeletal architecture during specific developmental stages.

## Materials and Methods

### Ethics Statement for Animal Experimentation

All animal procedures were carried out according to UK Home Office regulations and, protocols were approved by the University of Glasgow Ethics Committee (Project licence 60/2760).

### Molecular Cloning, Expression and Catalytic Activity of PPKL

Total RNA was isolated from gametocyte stage parasites and cDNA was generated using oligo(dTs) using the First Strand cDNA synthesis kit (Roche) according to manufacturer’s protocols. The catalytic domain was amplified using primers BamHI-GU935 and SacII-GU508 and cloned into a pet28a vector (Clontech). The catalytic site mutants were generated using primers GU1811 and GU1812 with a QuikChange Lightning mutagenesis kit. The plasmids were transformed into BL21(DE3)pLysS cells and 1L cultures were grown at 37°C to an O.D._600_ of 0.6 and induced with 1 mM IPTG at 30°C for 5 hours. Bacterial lystes were incubated with Nickel beads and protein was purified according to manufacturer’s protocols (Qiagen ) under native conditions. Elute was desalted using Zeba desalting columns (Pierce) and buffer exchanged into a 50 mM Tris, 100 mM NaCl and 10% glycerol solution. Purified recombinant protein was also used to generate rabbit antibodies (ProteinTech group) which were used further for western blots and immunofluorescence assays. Phosphatase assays were performed using EnzChek Protein phosphatase assay kit (Molecular Probes) with a modified reaction buffer. 1,2 or 5 ng of recombinant protein was incubated in 50 mM Tris-HCl at pH 7.0 containing 0.1 mM CaCl2, 125 µg/mL BSA, 0.05% Tween 20, 2 mM DTT, 20 mM MgCl_2_, 200 µM MnCl_2_ and 50 µM 6,8-difluoro-4-methylumbelliferyl phosphate (DiFMUP) for 30 minutes at 25°C. Fluorescence was read on a BMG FLUOstar Optima microplate reader at Ex/Em of 360/450 nm, and measurements were corrected for background fluorescence using a no phosphatase control.

### Generation of Transgenic Parasites

*Pbppkl* (*PBANKA_132950*) was targeted for genetic disruption by double-crossover homologous recombination with PL0035 vector containing the human dhfr/ts:yfcu selection cassette flanked by 1 kb targeting regions both upstream (KpnI-GU150, XhoI-GU151) and downstream (HindIII-GU148, SacII-GU149) of the ORF. Transfections and selection for mutant parasites was performed as published previously [49]. Primers used are shown in Table S2. *Pbppkl* was disrupted in 2 independent experiments and transgenic parasites were generated in both non-fluorescent background (*P. berghei* ANKA) and a wild type line which constitutively expresses a *gfp* transgene under the control of the *eef1a* promoter (*P. berghei* ANKA cl15cy1) [49] C-terminal GFP tagged PPKL parasite lines were generated using the PL0031 vector using single crossover homologous recombination containing 1 kb of the *ppkl* C-terminus amplified by using primers SacII-GU762 and BamHI-GU763. Correct integration of the targeting plasmid and gene disruption/modification was verified by Field inversion gel electrophoresis, diagnostic PCRs, Northern and Western analysis. Transgenic parasite lines were cloned by limiting dilution and used for phenotypic analysis.

### Phenotypic Analysis

Infections were performed by intraperitoneally injecting cryopreserved cloned parasites into Phenylhydrazine treated mice. Asexual parasites and proportion gametocytes were examined using giemsa stained smears. Exflagellation assays were performed 3 days post infection by adding 10 µl of infected blood into 300 µl of ookinete media (RPMI1640 containing 25 mM HEPES, 5 mM hypoxanthine, 20% FCS, 10 mM sodium bicarbonate, 100 µM xanthurenic acid at pH 7.6). 15 minutes post-activation, exflagellation centres were counted on a haemocytometer by light microscopy in 30 fields. Ookinete conversion was assessed 24 hours post-activation and growth in ookinete media. Conversion rates were calculated as percentage of mature ookinetes formed to macrogametes present. Fertility of macrogametes and microgametes was assessed by *in vitro* cross-fertilization assays where activated gametocytes were crossed with lines, which produce only fertile females (line137) or fertile males (line 270) [14]. Mosquito transmission experiments were performed by feeding 100 *Anopheles stephensi* for 15 minutes on anaesthesized infected mice (∼10% parasitaemia). Midguts were dissected 12–15 days post feeding and oocysts were counted on a Leica M205 FA fluorescence stereomicroscope using the GFP2 filter set. Images were taken by a Leica DFC340FX cooled monochrome digital camera. 17 days post-feeding salivary glands were dissected and imaged for sporozoites using the above microscope and camera system.

### Motility Assays

Ookinetes were collected 24 hours post activation and resuspended and embedded in 1∶1 ratio of RPMI and Matrigel™. 10 µl of sample was added on a glass slide and a cover-slip added and sealed with nail polish [17]. Samples were incubated for 1–2 hours at 21°C before imaging. Time-lapse movies were acquired every 20 seconds for 20 minutes on a Leica M205 FA fluorescence stereomicroscope. Motility paths were generated on Fiji software using the TOAST plugin [50].

### Scanning and Transmission Electron Microscopy

For SEM parasites were washed in PBS followed by fixation in 2% paraformaldehyde, 2% glutaraldehyde in 0,1 M sodium cacodylate buffer (pH 7.42) with added 0.1% magnesium chloride and 0.05% calcium chloride. After washes with sodium cacodylate buffer rinse (2% sucrose) with added chlorides, cells were further fixed in 1% osmium tetroxide in sodium cacodylate buffer. The samples were dehydrated via an alcohol series and solution exchanged with Hexamethyldisilazane, sputter coated with Au/Pd and imaged in a JSEM 6400. Parasites for TEM were fixed and processed as above till the alcohol dehydration. Specimens were treated with propylene oxide and embedded in Epon/Araldite resin mix. Resin sections (100 nm thick) were imaged with the Zeiss LEO 912 EFTEM.

### Immunofluorescence Assays

Dried smears were fixed with using 4% EM grade paraformaldehyde (in PBS). Fixed cells were washed twice in PBS and then permeabilized with 0.1% Triton X-100/PBS for 10 minutes, followed by PBS washes and blocked in 5% BSA/PBS for one hour. Samples were incubated with all primary antibodies at 1∶1000 dilution (in 5% BSA/PBS) except α-PPKL, which was at 1∶250 dilution either at 4°C O/N or for 2 hrs at room temperature. Cells were washed three times in PBS for 10 min each to remove excess primary antibody. Secondary antibody was added at 1∶3000 dilution (in 5% BSA/PBS) and allowed to bind for an hour. Cells were washed three times in PBS and mounted in Vectashield™ with DAPI. Images were examined on a DeltaVision Epifluorescence microscope (Applied Precision) under a ×100 objective and images were captured with a CoolSNAP HQ camera followed by deconvolution using SoftWorx.

### Western Blotting

For PPKL expression across the asexual life-cycle, parasites (gametocyte non-producer, *line 233*) were collected from one mouse at 5% parasetemia and O/N cultured in Schizont media (RPMI1640 containing 25 mM HEPES, 5 mM hypoxanthine, 20% FCS, 10 mM sodium bicarbonate). Mature schizonts were enriched on a 55% Nycodenz gradient and injected intravenously into 2 mice. Two hours post injection, infected blood was collected by cardiac puncture, leukocytes removed, and cultured in schizont media. Parasites were collected at appropriate time points and enriched by erythrocyte lysis. 10^7^ cells were lysed in RIPA buffer (50 mM TrisHCl pH 7.4, 150 mM NaCl, 2 mM EDTA, 1% NP-40, 0.1% SDS) supplemented with protease inhibitor (Roche) was used for analysis. Gametocytes were collected from sulphadiazine treated mice and purified on a 53% Nycodenz gradient. 10^6^ gametocytes and ookinetes generated from 10^6^ gametocytes were used for analysis. Clarified lysates were suspended in Laemmli sample buffer and separated on a 10% SDS-polyacrylamide gel. Samples were subsequently transferred to nitrocellulose membranes (Amersham Biosciences) and immunoblotting performed, probed with appropriate primary (1∶1000 in 5% milk/PBS) secondary antibodies (1∶5000 in 5% milk/PBS), and visualized with ECL kit (Pierce) or ECL Advance kit (Amersham). For the microneme secretion assay, 24 hr post activation, ookinetes (from a 5% gametocytemia culture with 70% ookinete conversion rate) were purified and on a LD50 magnetic column and incubated in PBS for 4 hours. The cells were spun down at 750×g and the supernatant collected, filtered through a 2 µm filter and concentrated on an Amicon-ultra centrifugal filter, 30 K cut-off (Millipore), mixed with Laemmli sample buffer and separated on a 4–15% Tris-HCL gradient gel (Biorad). Proteins transferred on nitrocellulose were probed α-CTRP (1∶5000) and α-Chitinase (1∶4000) and processed as detailed above.

### Bioinformatic Analysis

PPKL homologues were identified by searching the non-redundant protein sequences (nr) database with the complete PPKL protein sequence using Blastp (protein-protein BLAST). The top 34 sequences were aligned using ClustalX and the neighbour joining function was utilized to generate a boot-strapped tree with 1000 iterations.

### Statistics

All error bars shown in Figures are standard deviations of the mean (SD) and all p-values were determined by 2-tailed t-test and n-values are listed for biological replicates.

## Supporting Information

Figure S1 **Phylogenetic analysis of PPKL phosphatases.** The complete protein sequence of *P. berghei* PPKL was pBLAST searched in the NCBI non-redundant protein database and a rooted tree was generated with the top 34 hits. The sequences were aligned using ClustalX and the neighbour joining function was utilized to generate a boot-strapped tree (1000 iterations). Boot-strap values are indicated. Gene ids and description are reported in Table S1.
(TIF)

Figure S2 **Multiple sequence alignment of representative** ***Plasmodium*** **and** ***A. thaliana*** **PPKL catalytic domain and known phospho-sites.** A. The three signature motifs (-GDXHG-, -GDXVDRG and –GNHE-) of the PPP (Phosphoprotein Phosphatase) family are indicated in blue boxes; the catalytic residues are indicated as green hashes (#) and the five inserts in the catalytic domain which are specific to PPKLs are indicated with roman numerals (for more details on precise location of inserts see [21], [34]). B. Phospho-sites identified in [4] in PPKL protein during schizont stage. C. Comparison of phospho-sites in Schizonts [4] from *P. falciparum* (P.F.) and ookinetes (GeneDb) from *P. berghei* (P.B.). Corresponding amino-acid residues in the two species are shown with identified phospho-sites indicated in red.
(TIF)

Figure S3 **Generation of** ***ppkl^–^*** **and** ***ppkl-gfp*** **transgenic parasites.** A. Schematic representation of PL0035 *pbppkl* deletion vector, native and modified gene locus. The deletion vector contains 1 kb regions upstream and downstream of the gene ORF flanking a hdhfr/yfcu cassette.B. PCR showing successful integration and gene deletion. Primers used for diagnostic PCR are as follows 1: GU205/GU207, 2: GU204/GU206, 3: GU507/GU508. C. Field Inversion gel electrophoresis blot hybridized with *pbdhfr/ts* detects both the modified chromosome (13) and endogenous locus (7). D. Northern blot with 500 bp C-terminal probe confirms no *ppkl* RNA expression in mutant. E. Western blot analysis using α-PPKL polyclonal rabbit antibody raised against catalytic domain of endogenous protein shows no a ∼100 kDa band in *wt* (expected size of PPKL) protein expression in *ppkl^–^*. G. Schematic representation of *pbppkl-gfp* tagging vector, native and modified gene locus using a single crossover event where the gene was linearized using a BglII site. H. Diagnostic PCR confirming successful integration of the tagging sequence shown by following primers 1:GU532/GU147, 2:GU533/GU507. I. Field Inversion gel electrophoresis blot hybridized with *pbdhfr/ts* detects both the modified chromosome (13) and endogenous locus (7). J. Western blot analysis of ookinete lysate using α-GFP antibody detecting GFP (28 kDa) in a *wt* line and PPKL-GFP (∼130 kDa) in the transgenic line.
(TIF)

Figure S4 **Localization of PPKL in ookinetes and macrogametocyte.** A. PPKL-GFP shows similar localization in the ookinete compared to PPKL localization observed with polyclonal antibody against endogenous PPKL. B. PPKL expression in female gametocyte. DOZI (development of zygote inhibited):GFP line was used to examine female gametocyte specific expression [51] Bar = 2 µm. C. TEM images of longitudinal sections of various *ppkl^–^* ookinetes exhibiting elongated apical ends show varying degrees of abnormality. Bar = 1 µm.
(TIF)

Table S1 **List of Genes used for phylogenetic analysis in Fig. S1.**
(XLSX)

Table S2 **Primers used in study.**
(XLSX)

Video S1 **Mature** ***wt*** **ookinetes moving through Matrigel.**
(MOV)

Video S2 **Mature** ***ppkl^–^*** **ookinete exhibits negligible forward motion.**
(MOV)
