# Supplementary figures and images for: A Unique Kelch Domain Phosphatase in Plasmodium Regulates Ookinete Morphology, Motility and Invasion
Source: PLoS One. 2012 Sep 5;7(9):e44617. doi: 10.1371/journal.pone.0044617 (PMC3434153; doi:10.1371/journal.pone.0044617)

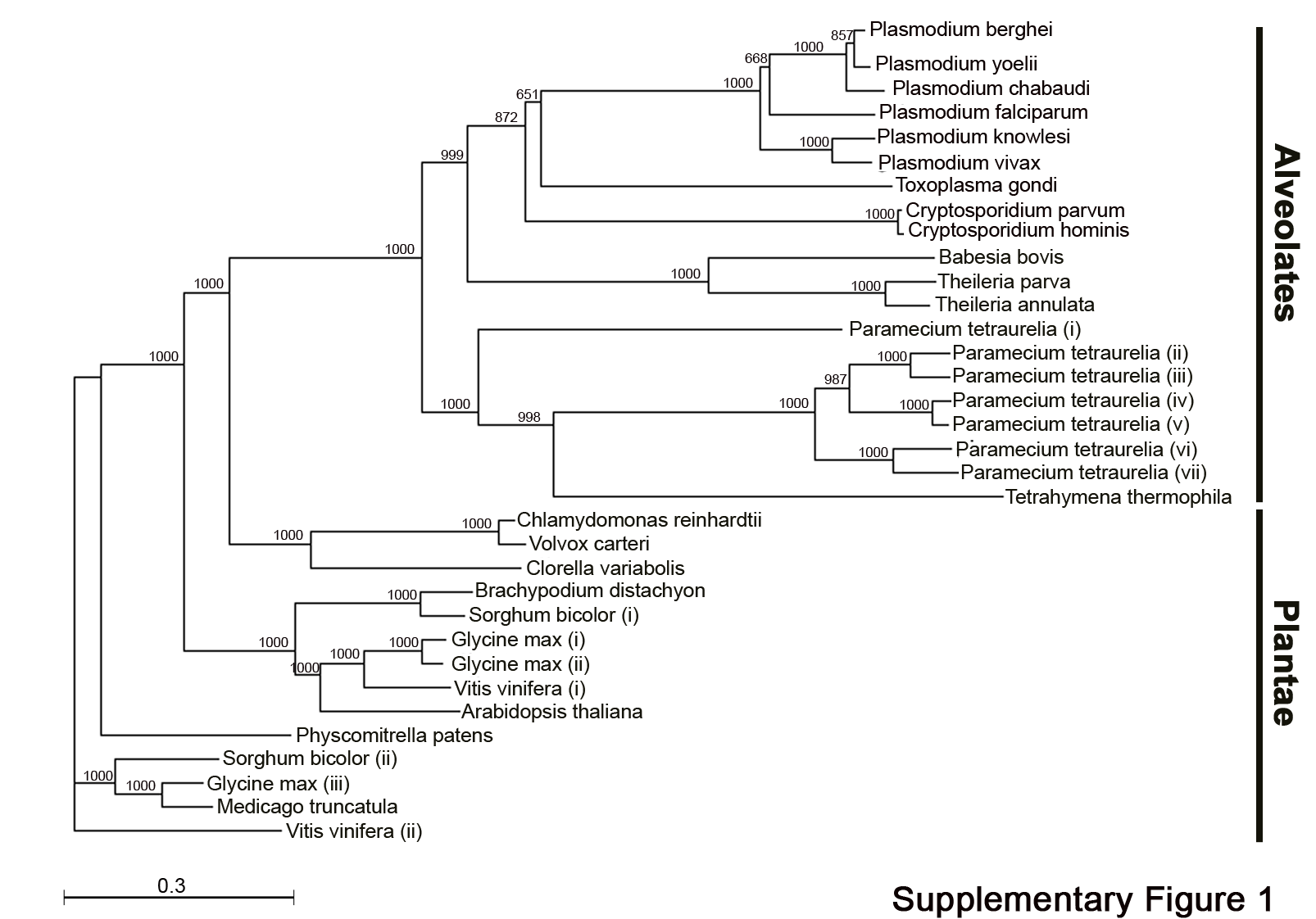

Supplement: Figure S1 — Phylogenetic analysis of PPKL phosphatases. The complete protein sequence of P. berghei PPKL was pBLAST searched in the NCBI non-redundant protein database and a rooted tree was generated with the top 34 hits. The sequences were aligned using ClustalX and the neighbour joining function was utilized to generate a boot-strapped tree (1000 iterations). Boot-strap values are indicated. Gene ids and description are reported in Table S1. (TIF) [file pone.0044617.s001.tif]

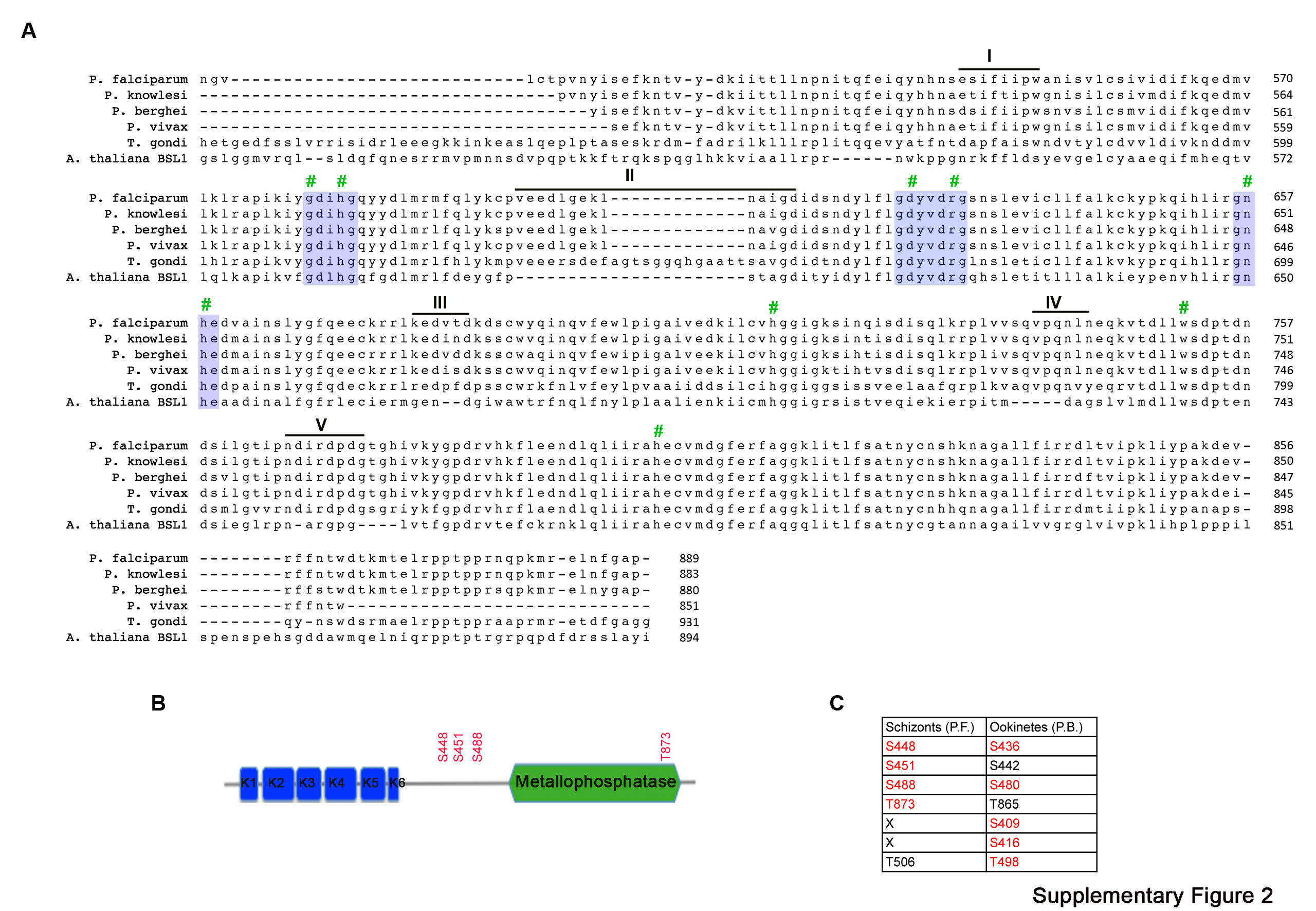

Supplement: Figure S2 — Multiple sequence alignment of representative Plasmodium and A. thaliana PPKL catalytic domain and known phospho-sites. A. The three signature motifs (-GDXHG-, -GDXVDRG and –GNHE-) of the PPP (Phosphoprotein Phosphatase) family are indicated in blue boxes; the catalytic residues are indicated as green hashes (#) and the five inserts in the catalytic domain which are specific to PPKLs are indicated with roman numerals (for more details on precise location of inserts see [21], [34]). B. Phospho-sites identified in [4] in PPKL protein during schizont stage. C. Comparison of phospho-sites in Schizonts [4] from P. falciparum (P.F.) and ookinetes (GeneDb) from P. berghei (P.B.). Corresponding amino-acid residues in the two species are shown with identified phospho-sites indicated in red. (TIF) [file pone.0044617.s002.tif]

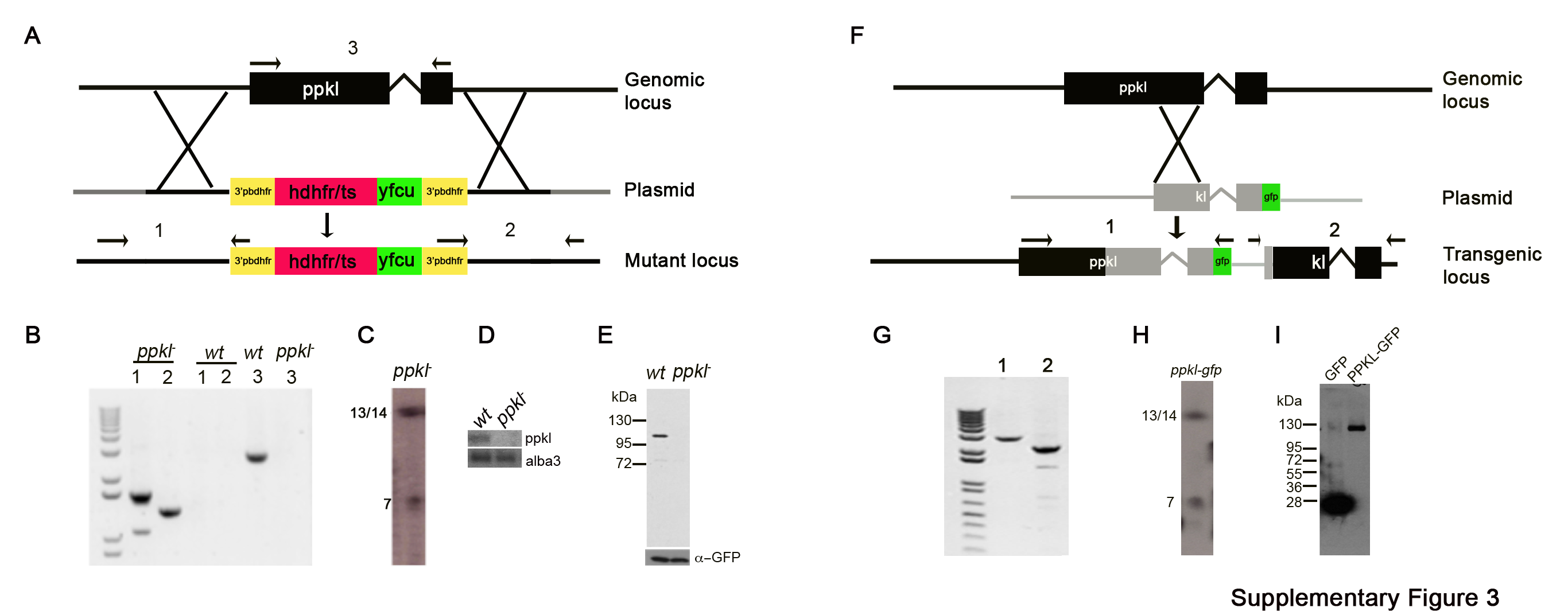

Supplement: Figure S3 — Generation of ppkl– and ppkl-gfp transgenic parasites. A. Schematic representation of PL0035 pbppkl deletion vector, native and modified gene locus. The deletion vector contains 1 kb regions upstream and downstream of the gene ORF flanking a hdhfr/yfcu cassette.B. PCR showing successful integration and gene deletion. Primers used for diagnostic PCR are as follows 1: GU205/GU207, 2: GU204/GU206, 3: GU507/GU508. C. Field Inversion gel electrophoresis blot hybridized with pbdhfr/ts detects both the modified chromosome (13) and endogenous locus (7). D. Northern blot with 500 bp C-terminal probe confirms no ppkl RNA expression in mutant. E. Western blot analysis using α-PPKL polyclonal rabbit antibody raised against catalytic domain of endogenous protein shows no a ∼100 kDa band in wt (expected size of PPKL) protein expression in ppkl–. G. Schematic representation of pbppkl-gfp tagging vector, native and modified gene locus using a single crossover event where the gene was linearized using a BglII site. H. Diagnostic PCR confirming successful integration of the tagging sequence shown by following primers 1:GU532/GU147, 2:GU533/GU507. I. Field Inversion gel electrophoresis blot hybridized with pbdhfr/ts detects both the modified chromosome (13) and endogenous locus (7). J. Western blot analysis of ookinete lysate using α-GFP antibody detecting GFP (28 kDa) in a wt line and PPKL-GFP (∼130 kDa) in the transgenic line. (TIF) [file pone.0044617.s003.tif]

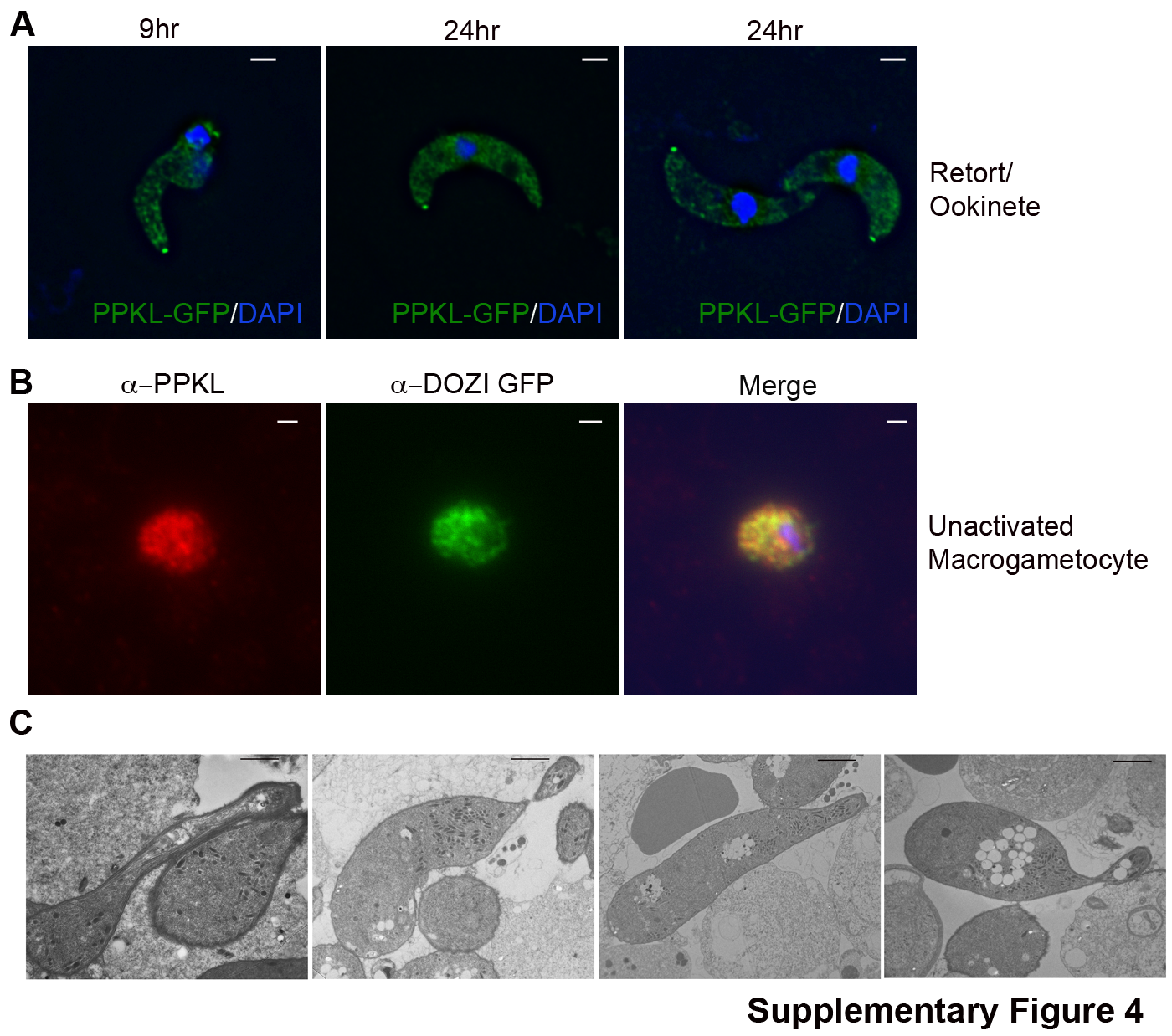

Supplement: Figure S4 — Localization of PPKL in ookinetes and macrogametocyte. A. PPKL-GFP shows similar localization in the ookinete compared to PPKL localization observed with polyclonal antibody against endogenous PPKL. B. PPKL expression in female gametocyte. DOZI (development of zygote inhibited):GFP line was used to examine female gametocyte specific expression [51] Bar = 2 µm. C. TEM images of longitudinal sections of various ppkl– ookinetes exhibiting elongated apical ends show varying degrees of abnormality. Bar = 1 µm. (TIF) [file pone.0044617.s004.tif]
